# Expression of Cathepsins B, D, and G in Infantile Hemangioma

**DOI:** 10.3389/fsurg.2015.00026

**Published:** 2015-06-17

**Authors:** Tinte Itinteang, Daria A. Chudakova, Jonathan C. Dunne, Paul F. Davis, Swee T. Tan

**Affiliations:** ^1^Gillies McIndoe Research Institute, Wellington, New Zealand; ^2^Centre for Biodiscovery, School of Biological Sciences, Victoria University of Wellington, Wellington, New Zealand; ^3^Centre for the Study & Treatment of Vascular Birthmarks, Wellington Regional Plastic, Maxillofacial and Burns Unit, Hutt Hospital, Wellington, New Zealand

**Keywords:** paracrine, renin–angiotensin system, infantile hemangioma, cathepsin, angiotensin converting enzyme

## Abstract

**Aims:**

The role of the renin–angiotensin system (RAS) in the biology of infantile hemangioma (IH) represents an emerging paradigm, particularly the involvement of renin, angiotensin converting enzyme, and angiotensin II. This study investigated the expression of cathepsins B, D, and G, enzymes that may modulate the RAS, in IH.

**Materials and Methods:**

The expression of cathepsins B, D, and G was examined using immunohistochemistry, enzyme activity assays, mass spectrometry, and NanoString gene expression assay in IH samples at different phases of development.

**Results:**

Immunohistochemical staining showed the expression of cathepsins B, D, and G in proliferating and involuted IH samples. This was confirmed at the transcriptional level using NanoString gene expression assays. Mass spectrometry confirmed the identification of cathepsins D and G in all three phases of IH development, whereas cathepsin B was detected in 2/2 proliferating and 1/2 involuting lesions. Enzyme activity assays demonstrated the activity of cathepsins B and D, but not G, in all phases of IH development.

**Conclusion:**

Our data demonstrated the presence of cathepsins B, D, and G in IH. Their role in modulating the RAS and the biology of IH offers potential novel targets for the management of this tumor.

## Introduction

Infantile hemangioma (IH) affects 4–10% of Caucasian infants (1–3). It typically undergoes rapid proliferation during infancy followed by spontaneous slow involution over 1–10 years, often leaving a fibro-fatty residuum (4, 5).

Recent data have implicated IH as an embryonic developmental anomaly due to aberrant proliferation and differentiation of a hemogenic endothelium (6) derived from a primitive mesoderm with a neural crest phenotype (7, 8). There is also evidence of a crucial role for the endocrine renin–angiotensin system (RAS) with the vasoactive peptide, angiotensin II (ATII), as a key regulator of this hemogenic endothelium (9–11). An understanding of the RAS pathway and the control mechanisms that govern the downstream production of the ATII (10) is critical in the understanding of the biology of this enigmatic condition and the observed efficacy of systemic administration of β-blockers (12) and angiotensin converting enzyme inhibitors (ACEi) (13) in the treatment of IH.

**Figure 1:**
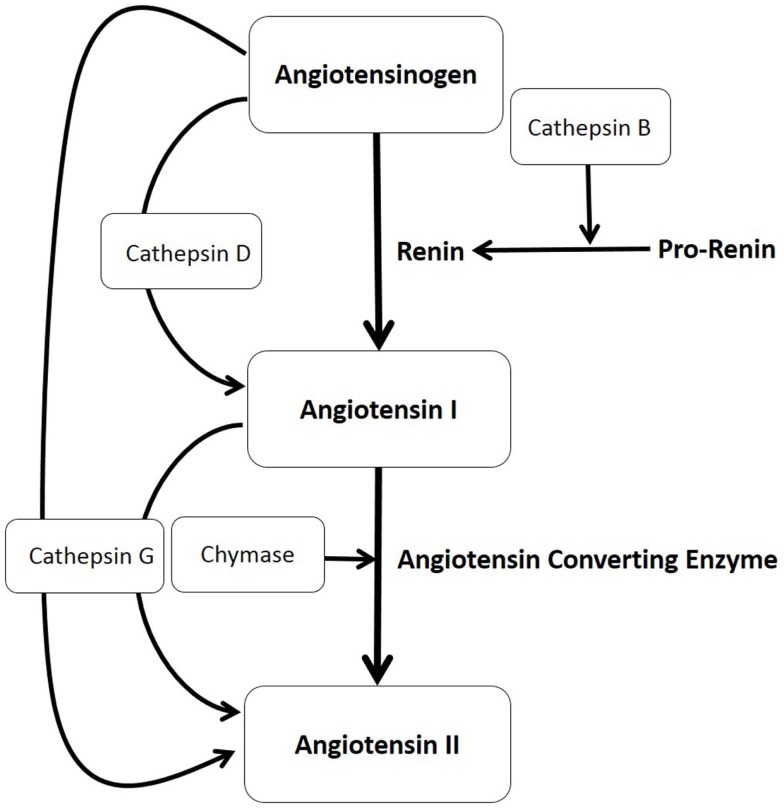
**The production of angiotensin II (ATII) involves the classical renin–angiotensin system (illustrated in bold letters) and paracrine bypass loops involving multiple enzymes (illustrated in unbold letters) in that angiotensinogen is converted to angiotensin I (ATI) either by cathepsin D or by renin, with renin converted from its precursor pro-renin form, by cathepsin B**. ATI is then cleaved to form ATII by either angiotensin converting enzyme, chymase, or cathepsin G, which also can directly convert angiotensinogen to ATII.

The classical RAS pathway involves the initial conversion of angiotensinogen to angiotensin I (ATI) by renin, with its subsequent conversion to ATII by angiotensin converting enzyme (ACE) (14) (Figure 1). Recent reports of the observed effect of topical β-blockers (15) and the variable effects of systemic β-blockers (16) and ACEi (13) for a given dosage on IH have led us to speculate on the possibility of non-classical RAS pathways acting as bypass mechanisms that contribute to the ultimate availability of ATII (17).

We have previously characterized the mast cells within IH and shown their abundant expression of chymase (18), thus providing evidence for the existence of one critical enzyme involved in a non-classical RAS pathway (Figure 1).

In this study, we investigated, within IH, the presence and localization of other non-renin and non-ACE pathways involving cathepsin B, an enzyme that converts pro-renin to active renin (19, 20); cathepsin D, a protease that converts angiotensinogen to ATI (21, 22); and cathepsin G, a protease with the ability to produce ATII from both angiotensinogen and ATI (19, 20) (Figure 1).

## Materials and Methods

Proliferating (*n* = 6), involuting (*n* = 3), and involuted (*n* = 6) IH samples were obtained from patients aged 4–7 months, 2–4 years, and 6–12 years, respectively, according to a protocol approved by the Central Health and Disability Ethics Committee (2 October 2013; Approval No: 13/CEN/130).

### Immunohistochemistry

Four μm-thick formalin-fixed paraffin-embedded IH sections of proliferating (*n* = 6) and involuted (*n* = 6) from 12 patients were used for immunohistochemical (IHC) staining. Antigen retrieval was performed using sodium citrate (Leica) at 95°C for 15 min. All sections underwent single 3,3-diaminobenzidine (DAB) staining for the primary antibodies, cathepsin B, 1:200 (Santa Cruz); cathepsin D, 1:200 (Leica); cathepsin G, 1:200 (Santa Cruz); tryptase, ready to use (Leica), CD34, ready to use (Leica); and GLUT-1, 1:200 (Cell Marque) with detection using the bond polymer refine detection kit (Leica). To confirm dual expression of two proteins, selected representative slides of each phase of IH underwent immuno-fluorescent (IF) IHC staining with the same primary antibodies at the same concentrations, but using an appropriate secondary antibody for detection (donkey anti-mouse Alexa-488 or donkey anti-rabbit Alexa 594, Life Technologies, NZ, USA). All antibodies were diluted in Bond primary antibody diluent (Leica), and all DAB and IF IHC staining were performed on the Leica Bond Rx auto-stainer (Leica, Australia). IF IHC stained slides were mounted using vectashield hardset medium with DAPI (Vector Laboratories, CA, USA).

### Mass spectrometry

Total protein extracted from proliferating (*n* = 2), involuting (*n* = 2), and involuted (*n* = 2) IH tissues from six patients of the original cohort was used for mass spectrometry. The tissues were homogenized in 150 μL of ice cold RIPA buffer (Sigma-Aldrich) containing 1× Complete Protease inhibitor cocktail EDTA-free (Roche Life Science) using a Teflon-coated dounce homogenizer. After protein quantitation (Qubit^®^ 2.0 Fluorometer, Life Technologies, San Diego, CA, USA), 100 mg of total protein from each sample was precipitated overnight at −20°C (ProteoExtract^®^ Protein Precipitation Kit, Merck Millipore). The washed protein pellets were re-suspended in 50 μL of 5% sodium deoxycholate (SDC), 10 mM dithiothreitol, 100 mM TEAB buffer (pH 8.5) (Sigma Aldrich), and incubated for 30 min at 80°C. Samples were then alkylated for 60 min in the dark using 40 mM iodoacetamide, and diluted to 500 μL final volume in 100 mM TEAB buffer (pH 8.5). Proteins were digested overnight at 37°C using 4 mg trypsin per sample (modified sequencing grade trypsin from bovine pancreas; Roche Life Science). SDC removal was achieved by formic acid precipitation (1% final volume) and centrifugation for 30 min at 13,000 × *g*, and the peptide supernatants transferred to fresh microcentrifuge tubes. The SDC precipitates were washed with 200 μL of 1% formic acid and each wash solution was combined with the previously recovered peptide supernatant. Samples were then concentrated to ~10 μL, reconstituted to 500 μL in 0.1% formic acid, purified using OMIX C18 pipette tips (Agilent Technologies), and prepared for liquid chromatography (LC)–MS/MS (mass spectrometry) in 4% acetonitrile: 0.1% formic acid.

Liquid chromatography–MS/MS (four technical replicates per sample) was performed using an UltiMate 3000 HPLC system (Dionex) connected to a LTQ Orbitrap XL mass spectrometer (Thermo Scientific). Thirty-five μL per sample per injection were loaded onto an Acclaim PepMap100, C18 column (3 μm, 100 Å, 75 μm i.d. ×15 cm, Thermo Scientific) (0.3 μL min^−1^ flow rate), and peptides were eluted and analyzed using data-dependent MS/MS acquisition (90 s exclusion window, top 8 peptides per MS scan selected for MS/MS). Raw MS/MS data files were searched against a complete human protein database (SwissProt KB, 22 October 2014, 69689 sequences) using Proteome DiscovererTM V1.4 (Thermo Scientific) and Scaffold 4.0 (Proteome Software) to establish protein identification and relative protein abundance by spectral counting. Peptide assignments were accepted above 90% confidence, and protein identification parameters were: protein threshold, 1.0% FDR; minimum total spectrum count, 2; peptide threshold, 1.0% FDR.

### Enzymatic activity

Enzymatic activities of cathepsins B, D, and G were determined fluorometrically in snap-frozen proliferating (*n* = 3), involuting (*n* = 3), and involuted (*n* = 3) IH samples from nine patients of the original cohort, using cathepsin B activity assay kit (Calbiochem), cathepsin D activity assay kit (Abcam), and cathepsin G activity assay kit (Abcam), respectively. All steps of the procedure were performed according to the manufacturers’ protocol. Fluorescence was measured in 96-well plate format using the Varioscan plate reader (ThermoFisher).

### NanoString analysis

Snap-frozen samples of proliferating (*n* = 6) and involuted (*n* = 6) IH from 12 patients were used to isolate total RNA for NanoString nCounter™ Gene Expression Assay (NanoString Technologies, Seattle, WA, USA). The RNA was extracted from frozen tissues using RNeasy Mini Kit (Qiagen) and quantitated by the NanoDrop 2000 Spectrophotometer (Thermo Scientific). RNA samples with A260/A280 ≥1.9 and A260/A230 ≥1.8 were subjected to the NanoString nCounter™ gene expression assay as performed by New Zealand Genomics Ltd. (Dunedin, New Zealand), according to the manufacturer’s protocol. Probes for the genes encoding cathepsin B (*CTSB*; NM_001908.3), cathepsin D (*CTSD*; NM_001909.3), cathepsin G (*CTSG*; NM_001911.2), and the housekeeping genes *CLTC* (NM_004859.2), *GUSB* (NM_00181.3), *HPRT1* (NM_00194.1), and *PGK1* (NM_000291.3) were manufactured by NanoString Technologies (Seattle, WA, USA). Raw data were analyzed by nSolver™ software (NanoString Technologies, Seattle, WA, USA) using standard settings and were normalized against the housekeeping genes.

### Image analysis

All confocal images were captured using the Olympus FV1200 confocal microscope (Tokyo, Japan). All bright field images were captured using the Olympus BX53 microscope fitted with an Olympus DP21 digital camera (Tokyo, Japan).

### Statistical analyses

Statistical analyses were performed using the Kruskal–Wallis one-way analysis of variance for independent samples, using IBM SPSS (Version 22). This assigns ranks to the data, with *p* < 0.05 considered statistically significant.

## Results

### Immunohistochemical staining

**Figure 2:**
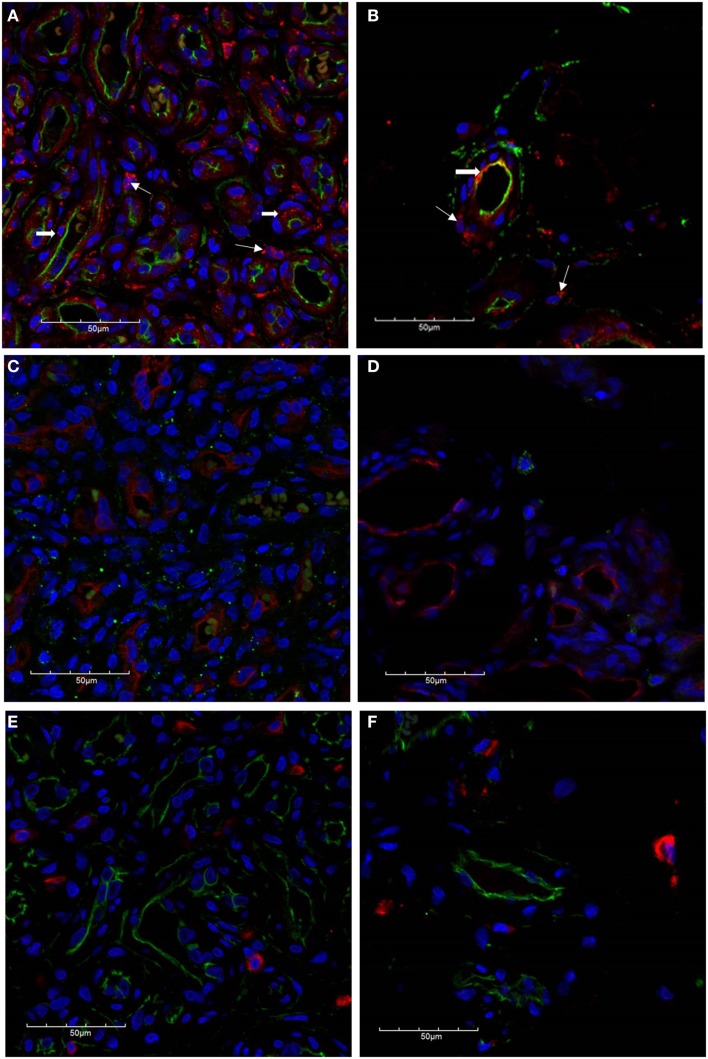
**Confocal immunofluorescent immunohistochemical images of proliferating (A, C, E) and involuted (B, D, F) IH samples**. The expression of cathepsin B [**(A,B)**, red] is seen on the endothelium [**(A,B)**, *thick arrows*] as well as cells in the interstitium [**(A,B)**, *thin arrows*] in proliferating and involuted IH. Cathepsin D was predominantly localized to cells of the interstitium [**(C,D)**, green] and appeared distinct from the endothelium expressing GLUT-1 [**(C,D)**, red]. Similarly, the expression of cathepsin G [**(E,F)**, red] was also restricted to cells of the interstitium and is independent of the CD34^+^ endothelium [**(E,F)**, green]. Cell nuclei [**(A–F)**, blue] are stained with 4′,6′-diamidino-2-phenylindole dilactate.

The microvessels of IH are composed of a distinct inner endothelial and a concentric outer pericyte layer (23, 24). All IH lesions used in the experiments were confirmed by the expression of GLUT-1 (data not shown), the marker used to differentiate IH from other vascular anomalies (23). The expression of cathepsin B (Figures 2A,B, red) was demonstrated in cells of the endothelium (Figures 2A,B, *thick arrows*), expressing CD34 (Figures 2A,B, green), as well as in the cells of the interstitium (Figures 2A,B, *thin arrows*), in both proliferating (Figure 2A) and involuted (Figure 2B) IH samples. This was in contrast to the expression of cathepsin D (Figures 2C,D, green), which was predominantly expressed by cells in the interstitium, away from the endothelium expressing GLUT-1 (Figures 2C,D, red) in proliferating (Figure 2C) and involuted (Figure 2D) IH. A similar staining pattern was also demonstrated for cathepsin G (Figures 2E,F, red) in cells in the interstitium, distinct from the endothelium expressing CD34 (Figures 2E,F, green), in both proliferating (Figure 2E) and involuted (Figure 2F) IH samples.

**Figure 3:**
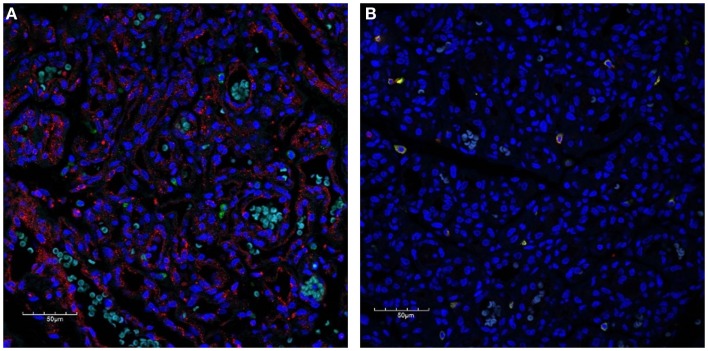
**Confocal immunofluorescent immunohistochemical images of proliferating IH showing distinct interstitial cellular populations expressing cathepsin B [red, (A)] and cathepsin D [green, (A)]**. Cathepsin G [red, **(B)**] was expressed by the phenotypic mast cells that also expressed tryptase [**(B)**, green)]. Cell nuclei [**(A,B)**, blue] are stained with 4′,6′-diamidino-2-phenylindole dilactate.

To further characterize the expression profile of the interstitial population expressing the cathepsins within proliferating IH, dual staining was performed for cathepsin B (Figure 3A, red) and cathepsin D (Figure 3A, green) confirming the expression by two distinct cellular populations. The expression of cathepsin G has previously been reported in mast cells (25). Using tryptase as a marker for mast cells, we performed dual staining for tryptase (Figure 3B, green) and cathepsin G (Figure 3B, red), which confirmed the expression of cathepsin G on the tryptase^+^ mast cells in the interstitium.

### Mass spectrometry

**Figure 4:**
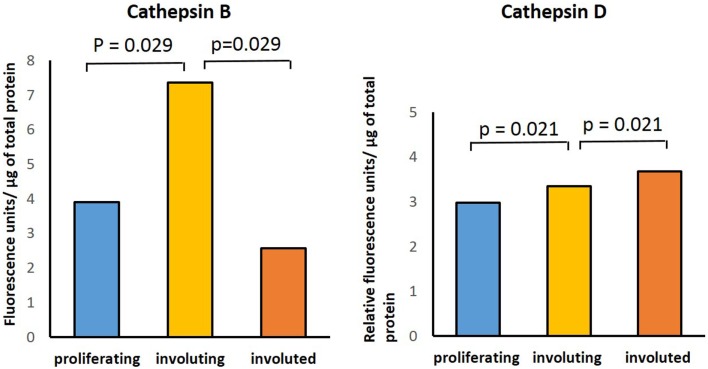
**Cathepsins B and D enzyme activity in infantile hemangioma tissues with enzyme activity assays performed in proliferating (*n* = 3), involuting (*n* = 3), and involuted (*n* = 3) samples**. The data were subjected to statistical analysis using the Kruskal–Wallis one-way analysis of variance for independent samples. *P* < 0.05 was considered as statistically significant.

Liquid chromatography–MS/MS analysis confirmed the presence of cathepsin B in proliferating and involuting IH tissues only, with cathepsins D and G identified throughout all three phases of IH. Spectral counting demonstrated that the relative abundance of cathepsin B was consistent between proliferating and involuting IH tissue (Figure 4), whereas cathepsins D and G remained relatively unchanged across all three phases. The protein identification values are summarized in Table S1 in Supplementary Material.

### Enzymatic activity

**Figure 5:**
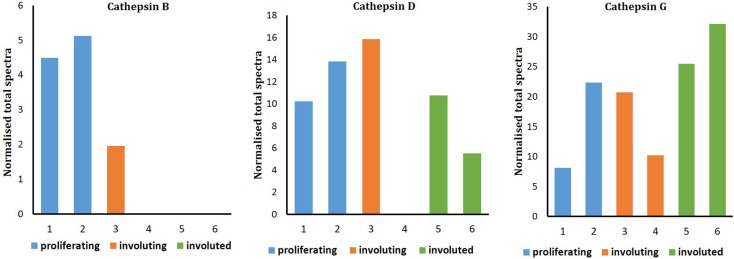
**Relative protein abundance of cathepsins B, D, and G extracted from proliferating (*n* = 2), involuting (*n* = 2), and involuted (*n* = 2) infantile hemangioma (IH)**. Proteome Discoverer™ V1.4 and Scaffold 4.0 were used to establish relative protein abundance by spectral counting. Relative protein abundance of all three cathepsins was unchanged between all three IH samples.

Enzymatic assays performed to determine the presence of activities for cathepsins B, D, and G in IH samples in all three phases of IH development confirmed the activity of both cathepsins B and D (Figure 5). Comparing the activity of cathepsin B across all three phases, there was statistical significance (*p* = 0.029) in the differences between the levels of activity in involuting compared to both proliferating and involuted IH samples used. Analysis of the enzyme activity of cathepsin D revealed statistically significant (*p* = 0.021) increase in the levels of activity from proliferating to involuting and from involuting to involuted phases of IH. Interestingly, the enzymatic activity of cathepsin G was barely detectable in all IH tissues examined (data not shown).

### NanoString assay

**Figure 6:**
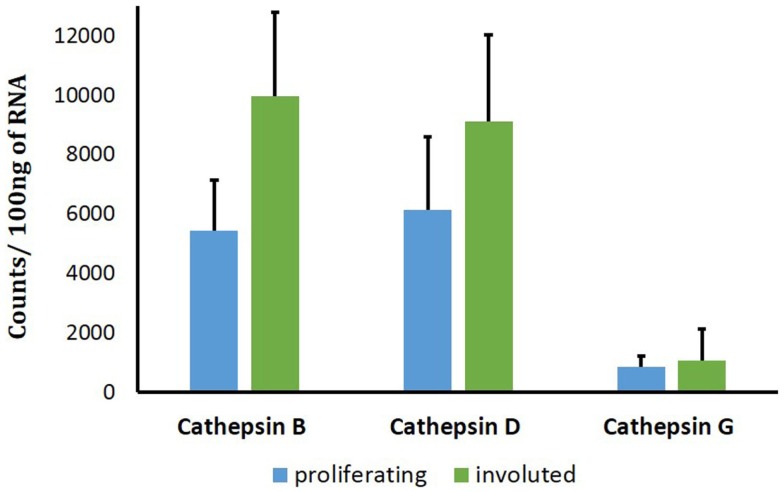
**Cathepsins B, D, and G mRNA levels in infantile hemangioma (IH) tissues**. NanoString nCounter Gene Expression assay with specific probes for *CTHB*, *CTHG*, and *CTHD* genes was performed using proliferating (*n* = 6) and involuted (*n* = 6) IH tissue samples. The data are presented as mean ± SD, and statistical analyses showed no significance.

To support the translational abundance of cathepsins B, D, and G examined in this study, we used transcriptional profiling of the tissue samples for corresponding levels of mRNA at all phases of IH development. The levels of mRNA for both cathepsins B and D were relatively high. This was in comparison with significantly lower levels for cathepsin G mRNA (Figure 6). No statistically significant differences of cathepsin D and cathepsin G mRNA levels were observed between proliferating and involuted IH samples. However, for cathepsin B, the involuted IH samples expressed significantly higher (*p* = 0.007) amounts of corresponding mRNA compared to proliferating samples.

## Discussion

We have recently demonstrated the crucial role for ATII in the biology of IH (10). ATII, a vasoactive peptide, is the downstream product of the RAS pathway, which in part accounts for the programed biological behavior of IH (9, 26) and the observed efficacy of the systemic administration of β-blockers (9) and captopril, an ACEi (13).

More recent reports have shown variable effects of systemic β-blockers for a given dosage (16) on IH. However, it is not possible to judge from the reports the traits of the slow responding lesions, as to whether location, multiplicity, or the size/volume of the lesions are determining factors for the relative responses. We have also observed variable effects of low-dose captopril on proliferating IH in patients for a given dosage (13). This may be due to the relatively low dosage of captopril used in the trial with possible spill-over of production of the downstream ATII, to innately high circulating of renin, or potential existence of paracrine, non-classical, RAS bypass pathways (Figure 1) (17). The latter possibility forms the basis of this investigation.

The final production of ATII results from the classical RAS pathway that depends on the presence of both renin and ACE but also the non-renin/non-ACE pathways involving a number of proteases (17, 27). Chymase, an enzyme critical for the conversion of ATI to ATII (Figure 1) (28) has previously been demonstrated to be expressed by the mast cells within IH (18). These phenotypic mast cells have been more recently identified to possess a primitive myeloid phenotype by their expression of the stem cell marker, Nanog, in the proliferating and involuting, but not involuted IH (29).

Our finding of the presence of cathepsins B, D, and G at both transcriptional and translational levels within IH, and the previous demonstration of the abundance of chymase (18) and ACE (9) suggests a system primed for downstream ATII production (Figure 1). It is intriguing that, although we have detected the presence of cathepsin G at both the transcriptional and translational levels, the enzymatic activity was not significant. It is possible that the presence of inhibitors of cathepsin G, such as serpins (30), may contribute to this apparent inconsistency, which is currently the topic of further investigation. We were unable to detect the presence of cathepsin B in the involuted IH samples by mass spectrometry, despite its presence being detected on IHC staining and high transcriptional levels. We infer a sampling error and/or low sample numbers leading to the inability to detect it rather than its absence. Our initial model of the classical RAS (31), with the high levels of circulating renin, presumed that IH has access to both the circulating endocrine angiotensinogen and ATI peptides (Figure 1). However, multiple paracrine proteases demonstrated in this study may also promote the production of ATII. This provides potential bypass mechanisms for IH to produce ATII that potentially promotes tumor growth (14), despite β-blockade or ACE inhibition (Figure 1).

The presence of cathepsins B, D, and G and chymase offers possible explanations that certain lesions are relatively more refractory to β-blockade and ACE inhibition. ACE inhibition results in an accumulation of ATI, which can potentially be converted to ATII by chymase produced by mast cells. This study demonstrates the presence of potential shunting/shuffling of upstream precursor peptides, through these non-classical RAS pathways, which we infer, may subsequently play a more prominent role once the classical RAS undergoes blockade.

This report reveals the expression of cathepsins B, D, and G in IH and offers novel insights into their roles in the RAS pathway, in this tumor. It provides understanding of the potential multiple pathways contributing to the ultimate levels of the vasoactive peptide, ATII, and highlights the complexity of β-blockade or ACE inhibition in the treatment of this tumor. Further investigation of the functional roles of cathepsins B, D, and G, and chymase, in the complex interplay of enzymes involved in the RAS pathway is needed to unravel their precise role of RAS in the biology of IH.

## Key Messages

1. This report highlights the presence of cathepsins B, D, and G in infantile hemangioma (IH).
2. Cathepsins B, D, and G within IH may play a crucial role in the production of angiotensin peptides in proliferating lH.
3. The presence of potential bypass mechanisms for the production of angiotensin peptides highlights the role for the RAS in proliferating IH.

## Author Contributions

TI, PFD, and STT performed the basis of the research. TI, PFD, and STT designed the research study. TI performed the IHC experiments and analysis. DAC performed enzyme activity experiments and analysis of the enzyme activity and NanoString data. JCD performed the mass spectrometry experiments and analysis. TI and STT drafted the manuscript. All authors contributed to the preparation of this manuscript. All authors read and approved the manuscript.

## Conflict of Interest Statement

There was no source of funding for the article. The authors declare that there is no source of financial or other support, or any financial or professional relationships, which may pose a competing interest. The content of this article has not been submitted or published elsewhere.

## Supplementary Material

The Supplementary Material for this article can be found online at http://journal.frontiersin.org/article/10.3389/fsurg.2015.00026
