# Supplementary material for: Expression of Cathepsins B, D, and G in Infantile Hemangioma
Source: Front Surg. 2015 Jun 17;2:26. doi: 10.3389/fsurg.2015.00026 (PMC4470331; doi:10.3389/fsurg.2015.00026)
Supplement: Supplementary file 1 [file table_1.pdf]

Supplementary Table 1 Scaffold V4.0 Protein Identification Values for Cathepsins B, D, and G in Proliferating, Involuting and Involuted Infantile Hemangioma Tissue Protein Extracts

	Cathepsin	Protein ID probability[1]	% Cov.	No. of Unique peptides[2]	No. of Peptides	No. of PSMs	% of total spectra
**Proliferating** (n=2)	B	100%<br>100%	10<br>5.3	2/1	2/1	6/7	0.013<br>0.010
	D	100%<br>100%	15<br>25	2/3	4/6	14/19	0.030<br>0.032
	G	100%<br>100%	25<br>42	4/9	4/9	11/31	0.023<br>0.052
**Involuting** (n=2)	B	92%<br>NI	5.3<br>NI	1/NI	1/NI	2/NI	0.0036<br>NI
	D	100%<br>NI	18<br>NI	2/NI	5/NI	16/NI	0.028<br>NI
	G	100%<br>100%	38<br>26	6/4	6/4	21/9	0.037<br>0.016
**Involuted** (n=2)	B	57%<br>NI	5.3<br>NI	1/NI	1/NI	1/NI	0.0023<br>NI
	D	100%<br>100%	15<br>6.8	2/2	4/2	11/2	0.025<br>0.0051
	G	100%<br>100%	41<br>42	7/6	7/6	26/12	0.059<br>0.031

[1]Protein identification parameters: Protein threshold, 1.0% FDR; Minimum total spectrum count, 2; Peptide threshold, 1.0% FDR. NI, protein not identified.
[2]Scaffold minimum peptide identification probability, >90%.
